# The Predictive Role of Grapho-Morphological Knowledge in Reading Comprehension for Beginning-Level L2 Chinese Learners

**DOI:** 10.3389/fpsyg.2021.757934

**Published:** 2021-11-11

**Authors:** Tianxu Chen, Sihui Ke, Keiko Koda

**Affiliations:** ^1^College of International Education, Minzu University of China, Beijing, China; ^2^Department of Modern and Classical Languages, Literatures and Cultures, University of Kentucky, Lexington, KY, United States; ^3^Department of Modern Languages, Carnegie Mellon University, Pittsburgh, PA, United States

**Keywords:** grapho-morphological knowledge, reading comprehension, L2 Chinese, adult, radicals

## Abstract

Reading comprehension entails a set of distinct, yet interdependent cognitive, linguistic, and nonlinguistic processes. Previous second language (L2) Chinese studies have identified significant and positive impacts of grapho-morphological knowledge at the character and subcharacter (radical) levels on passage reading comprehension; however, little is known regarding how early L2 grapho-morphological knowledge at the character and radical levels jointly predict later L2 reading comprehension. This study aimed to fill this gap. One hundred and five beginning-level L2 Chinese collegiate learners were recruited, and completed two character-related and two radical-related tasks in Week 8, as well as one reading comprehension tasks in Week 18. The main findings, based on correlational and path analyses, suggested that L2 Chinese learners’ early character-level and radical-level grapho-morphological knowledge significantly predicted later reading comprehension, yet the interrelations among grapho-morphological knowledge at the character and radical levels were complex. Path analyses identified direct and indirect paths from early character-level grapho-morphological knowledge to later reading comprehension, as well as indirect paths from early radical-level grapho-morphological knowledge to later reading comprehension. Methodological and pedagogical implications for L2 Chinese reading research and practices are discussed.

## Introduction

According to the componential view of reading, reading comprehension entails a set of distinct, yet interdependent cognitive, linguistic, and nonlinguistic skills from the lower levels to higher levels (Carr and Levy, 1990; Koda, 2005), among which grapho-morphological knowledge plays an important role in both first language (L1) and second language (L2) reading (Kirby and Bowers, 2017). Grapho-morphological knowledge is defined as “the ability to reflect upon how semantic information is encoded in the orthography and how orthography provides cues to meaning” (Kuo and Anderson, 2008, p.54). It should be noted that grapho-morphological *knowledge* has also been termed as grapho-morphological *awareness* or grapho-morphological *processing*, referring to the more explicit versus more implicit/tacit processes in which readers manipulating the mapping among morphology, phonology and orthography in a word. Following Nagy et al. (2014), we have adopted “knowledge” as an overarching umbrella term in this study.

Previous cross-sectional studies have identified the positive effects of grapho-morphological knowledge on reading comprehension among L1 alphabetic (English) speakers (e.g., Levesque et al., 2017), L1 non-alphabetic (Chinese) speakers (e.g., Ku and Anderson, 2003; Tong et al., 2009; Zhang, 2017), Chinese-English bilingual children (e.g., Pasquarella et al., 2011), school-aged Chinese-speaking learners of English as a Foreign Language (e.g., Zhang and Koda, 2013), and American university Chinese heritage language learners (e.g., Zhang and Koda, 2018). Recently, a few researchers have investigated how grapho-morphological knowledge acquired during spoken language development at the early stage of learning a language (abbreviated as *early* in this study) predict literacy development among native Chinese-speaking children (e.g., Tong et al., 2011; Pan et al., 2016). Compared against cross-sectional studies based on correlational and observational evidence, longitudinal research is advantageous for it provides inferences about the causal relationship between grapho-morphological knowledge and reading development (see a review in Ke and Zhang, 2021). However, little is known regarding the longitudinal relationship between early L2 grapho-morphological knowledge and later reading comprehension in L2 reading development during a relatively long period. *Later* means a different point in time after the early period of learning a language (cf. Ortega and Iberri-Shea, 2005, p.32).

This study aimed to uncover how early character- and radical-level grapho-morphological knowledge jointly predicts reading comprehension in L2 Chinese in beginning-level collegiate learners. It is expected that it will provide theoretical, methodological, and pedagogical implications for L2 reading research and practices.

### The Unique Grapho-Morphological Writing System of Chinese

Unlike the writing systems of languages with alphabetic scripts (e.g., English), Chinese has a unique multilevel writing system based on characters (Taft et al., 1999). Specifically, the orthographic units can be divided into four levels: multicharacters, characters, radicals, and strokes. The corresponding semantic units can be categorized as multicharacters, characters, and semantic radicals. Therefore, the Chinese grapho-morphological system is divided into character and radical levels (Shu and Anderson, 1997; Li et al., 2002; Wu et al., 2009). At the character level, a Chinese character is mostly (more than 93%) treated as a single morpheme (Yuan and Huang, 1998), which refers to a minimal, linguistic unit combining a meaning or grammatical function with a sound (Finegan, 2007). Single characters or multicharacters can form words in Chinese. At the radical level, compound characters’ grapho-morphological features are indicated by connections between semantic radicals’ forms and meanings in Chinese (Wu et al., 2009). In fact, most (80–90%) characters in modern Chinese are semantic-phonetic compound characters (Shu, 2003), within which a semantic component (*radical* in this study) offers information on the semantic category and a phonetic component provides a clue to the pronunciation. Compound characters sharing an identical semantic radical represent a similar semantic category. For example, the characters 湖 [(xu) (35), “lake”], 河 [(xɤ) (35), “river”], and 池 [(tʂʰ) (35), “pond”] have the same radical 氵 (water): thus, the meanings of these characters all relate to the meaning of 水 [(ʂu̯eɪ̯) (214), “water”]. Chinese pronunciation was transcribed into IPA following Handel et al. (2021). The major function of semantic radicals thus is to distinguish a large number of homophones, such as 晴, 情, 氰, based on their meanings. The distinctive characteristics of the Chinese writing system, then, mean that grapho-morphological knowledge is extremely important in reading Chinese both for native Chinese speakers and L2 Chinese readers.

### The Role of Grapho-Morphological Knowledge in L1 and L2 Chinese Reading Comprehension

It has been generally accepted that grapho-morphological knowledge plays an important role in reading comprehension for native Chinese speakers (e.g., Ku and Anderson, 2003; Shu et al., 2006; Zhang et al., 2012; Yeung et al., 2013; Zhang, 2017). For example, Shu et al. (2006) found that the ability to identify, analyze, and manipulate morphemes in words was a strong, consistent predictor of literacy-related skills (e.g., character reading and reading comprehension) among 75 Chinese children with reading difficulties and 77 without reading difficulties in the 5th and 6th grade. In addition, Zhang et al. (2012) investigated the relationship among morphological awareness (broadly defined as learners’ sensitivity to word-internal structures) at the word-level, orthography-semantic awareness at the radical level, and reading comprehension among 164 Hong Kong Chinese primary school students. They found that grapho-morphological knowledge at the character and radical levels (i.e., morphological awareness and orthography-semantic awareness) broadly and uniquely explained reading comprehension beyond word recognition and phonological processing skills.

Regarding the contribution of character-level grapho-morphological knowledge to L2 Chinese reading comprehension, a few empirical studies have recently investigated the mediated relationships among grapho-morphological awareness, vocabulary knowledge, and reading comprehension in L2 Chinese (e.g., Wu, 2017; Zhang and Koda, 2018). For example, Zhang and Koda (2018) explored the role of grapho-morphological awareness in L2 reading ability among 195 English-speaking adult learners of Chinese as a heritage language. One of their findings showed that grapho-morphological awareness significantly contributed to reading comprehension directly, independent of vocabulary knowledge. Similarly, Wu (2017) investigated the relationship between grapho-morphological awareness and reading comprehension in L2 Chinese among 143 intermediate-level Thai-speaking adult Chinese language learners. Wu’s study found that both grapho-morphological awareness and vocabulary knowledge had positive effects on L2 Chinese reading comprehension. Nevertheless, both studies merely focused on grapho-morphological knowledge at the character level, not examining radical-level knowledge.

Although substantial research has supported that grapho-morphological knowledge at the radical level facilitated single-character and multicharacter word reading (e.g., Tong and Yip, 2015; Wong, 2017), few L2 Chinese studies, to our knowledge, investigated the role of this knowledge in reading comprehension. Ke and Chan (2017) investigated L2 Chinese reading strategies by a think-aloud method among 68 L2 Chinese learners of three different proficiency levels in China. Their qualitative analyses indicated that L2 Chinese readers prefer to use bottom-up strategies, such as using information of a familiar radical. Yang (2020) analyzed word reading and reading comprehension among 70 L2 Chinese learners from American universities and found that orthographic and morphological factors were main types of reading errors. The two studies reviewed above seemed to support the positive role of radical-level grapho-morphological knowledge in L2 Chinese reading comprehension, but neither directly measured L2 Chinese radical-level grapho-morphological knowledge.

### Early Grapho-Morphological Knowledge in Later Reading Comprehension in Chinese

Although a few studies have discussed the relations between early grapho-morphological knowledge and later reading comprehension in an alphabetic language (i.e., English) as L1 or L2 (e.g., Deacon and Kirby, 2004; Deacon et al., 2014), evidence in Chinese has just emerged recently. Among L1 Chinese studies, it has been found that grapho-morphological knowledge plays a unique role in word reading (e.g., Tong et al., 2011; Yeung et al., 2013) and reading comprehension longitudinally (e.g., Pan et al., 2016; Zhang, 2016). For example, Pan et al. (2016) investigated 294 Chinese-speaking children in an 8-year longitudinal study. One main finding showed that preliterate phonological and morphological awareness at ages 4 to 6 indirectly affected character reading and reading comprehension at age 11, through grapho-morphological awareness at ages 7 to 10. The result emphasized the importance of the possible indirect contribution of early grapho-morphological knowledge to later reading comprehension.

Viewed collectively, grapho-morphological knowledge plays an important role in reading Chinese, which adopts a morphosyllabary writing system. Although a few empirical cross-sectional studies have provided evidence supporting the positive effects of grapho-morphological knowledge in L2 Chinese reading comprehension, there is still a need for a comprehensive investigation that measures grapho-morphological knowledge at character and radical levels and examines how character- and radical level grapho-morphological knowledge contributes to later L2 Chinese reading comprehension from a longitudinal perspective.

### Research Questions and Hypotheses

This study focused on the relationship among early grapho-morphological knowledge at the character level (operationalized as character recognition and manipulation) and at the radical level (operationalized as radical identification and analysis), as well as later reading comprehension for beginning-level L2 Chinese collegiate learners.

**FIGURE 1:**
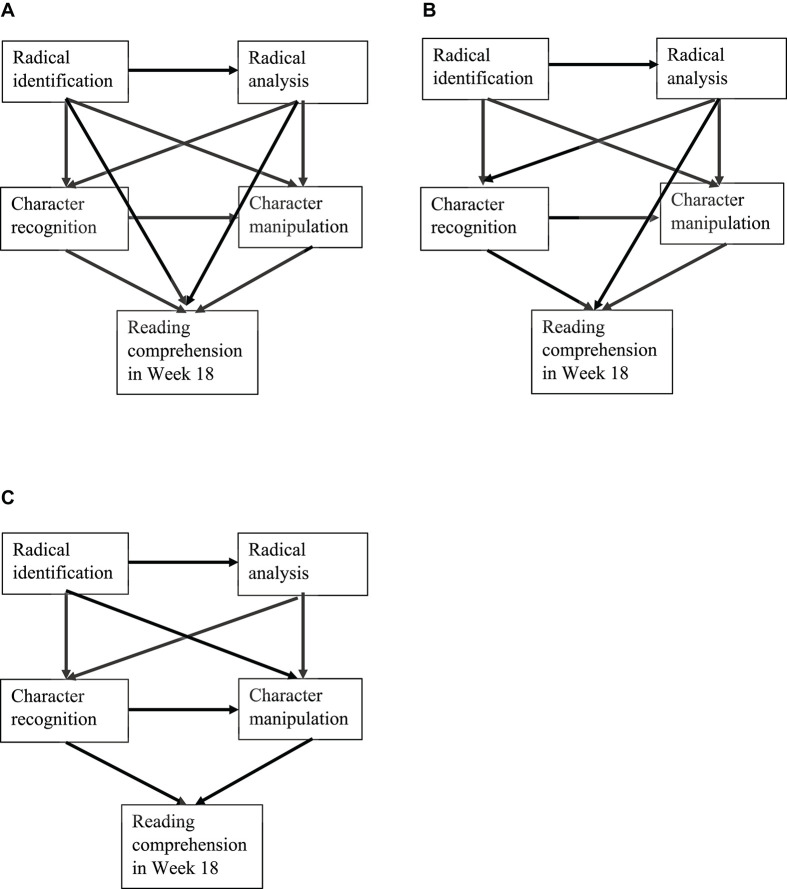
Three different conceptual models **(A–C)** of the interrelationships among early radical-level and character-level grapho-morphological knowledge as well as later reading comprehension.

Grounded in existing literature of reading research (e.g., Shen and Ke, 2007; Lü et al., 2015; Pan et al., 2016; Zhang and Koda, 2018), it was hypothesized that (1) early grapho-morphological knowledge at both character and radical levels significantly predict later reading comprehension in L2 Chinese; and (2) early character-level grapho-morphological knowledge and early radical-level grapho-morphological knowledge, to varying degrees, directly and indirectly predict later reading comprehension (see the conceptual models in Figures 1A–C). The hypotheses were translated into one guided research question: *What are the direct and indirect contributions of early character-level grapho-morphological knowledge and early radical-level grapho-morphological knowledge to later reading comprehension in L2 Chinese?*

## Materials and Methods

### Participants

A total of 105 beginning-level L2 Chinese learners at one university in China took part in this study (45 females and 60 males). Their ages ranged from 18 to 20 years old, and they came from 40 countries. All of the participants self-reported their L1 or official language to be a language with an alphabetic script, like English, Spanish, Russian, and French. All participants were enrolled in the Beginning-I Chinese program, in which the participants received intensive Chinese instructions (20 h per week) and used the textbook *Chinese in 10 Days* (Yang, 2016). They were taught by different instructors, yet they used the same teaching syllabus in the same Chinese program. None of the participants had visited China nor studied Chinese before, which means that they did not have any listening, speaking, reading or writing skills in Chinese when they began the Chinese courses in Week 1 (the beginning of the semester). During Week 1 and Week 2 of the Beginning-I Chinese course, all participants learned Pinyin (the Romanization script of the Chinese characters based on their pronunciation in Mandarin Chinese) and were given brief introductions about characters in the form of basic knowledge and rules about strokes and radicals.

### Measures

The study’s measures were all based on paper-pencil tasks, including character recognition, character manipulation, radical identification, radical analysis, and reading comprehension. The task scores were used to represent the participants’ corresponding L2 Chinese reading abilities. It is predicted that a participant with a stronger ability should perform better and receive a higher score on the corresponding task.

#### Radical Identification Task

This task was adapted from Chen (2019), and it specifically investigated the participants’ orthographic ability to identify the semantic radical in a compound character. The participants were presented with a low-frequency unfamiliar compound character, like 熨 [(yn) (51), “ironing”] and four Chinese options, like 火 [(xuɔ) (214), “fire”], 尸 [(ʂʅ) (55), “dead body”], 示 [(ʂʅ) (51), “to show”], and 寸 [(tsʰuən.) (51), “1/3 decimeter; short”]. Among these options, each represents one component in the target character. The participants were asked to circle the radical indicating the meaning of the character. In this case, the correct answer was 火. There were 24 target items, including “睅, 蒯, 魇, 徼, 罴, 绺, 铏, 圛, 馓, 𢌫, 隩, 邂, 愬, 籁, 愀, 屦, 猢, 熨, 凛, 窿, 禊, 霭, 膻, 赘” Each correct response was calculated as one point, and the maximum score in this task was 24. The Split-Half reliability of the task was excellent (Cronbach’s α = 0.90).

#### Radical Analysis Task

This task measured the participants’ ability to identify semantic radicals within a compound character and analyze whether the semantic radical is related to the character’s meaning. Twenty commonly used radicals were selected from the participants’ textbooks based on their radical familiarity, including “阝, 火, 冫, 饣, 穴, 辶, 雨, 彳, 贝, 廴, 犭, 钅, 忄, 灬, 刂, 广, 目, 竹, 囗, 纟.” The mean of radical familiarity was 3.20 out of 4, which was based on statistics from Lü et al. (2015, p. 6). All of the selected characters were complex characters (the mean of stroke numbers was 16.67). A picture and four very low-frequency unfamiliar compound characters were presented (see details in Table 1 below). The participants were asked to circle the character that best describes the picture. In this case, the expected answer was Option-2. Each correct response was calculated as one point, and the maximum score was 20 points. The Split-Half reliability of the task was acceptable (Cronbach’s α = 0.71).

**TABLE 1:** An example of the radical analysis task.

Target picture	
	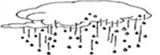
Characters pronunciation characters’ meanings	
Option-1 曥 [lu] (35) [colors of sun]	
Option-2 霺 [uei] (55) [light rain]	
Option-3 攕 [iεn] (55) [tender (for describing hands)]	
Option-4 幮 [tʂʰu] (214) [cloth]	

*Pronunciation and characters’ meanings were not provided in the real task.*

#### Character Recognition Task

This task measured the learners’ ability to recognize familiar characters from graphic forms. The task was adapted from Liu (2013), which asked the participants to indicate (1) their familiarity with 40 characters from the participants’ textbooks (learned and will learn) by circling “Yes” or “No” and (2) the sounds of the characters by writing down their Pinyin. The participants were not required to guess characters’ sounds when they were unsure. Each “Yes” selection with correct Pinyin was calculated as one point, and the maximum score for this task was 40 points. We did not record individual participants’ audio responses for two reasons: (1) to avoid penalizing L1 alphabetic background participants’ non-native speech or tone mistakes; (2) to make the task feasible for group administration. The Split-Half reliability of the task was good (Cronbach’s α = 0.85).

#### Character Manipulation Task

The format of this task was adopted from Wu et al. (2009). The character manipulation task measured the participants’ ability to manipulate multiple characters to produce compound words. Twenty-five high-frequency characters were provided in a table, including “每, 人, 天, 女, 水, 高, 店, 上, 大, 美, 小, 明, 酒, 家, 客, 工, 来, 本, 下, 回, 生, 子, 起, 国, 日.” All these characters were familiar to the participants. The participants were asked to create as many new words as possible in 5 min using the 25 characters. For example, the character “每” [(memeɪ̯) (214), “eery”] and the character “人” [(ʐən) (35), “person”] can be composed as the word “每人” [(memeɪ̯) (214) (ʐən) (35), “everyone”]. This study used total numbers of real words that the participants created to represent their character manipulation ability. The criteria of a word were based on the Committee of Contemporary Chinese Dictionary (2003). It should be noted that the 25 characters in the task could compose 60 two-character words in the Chinese dictionary, but many possible composed words are unfamiliar for L2 learners, such as 本家 or 本家. Therefore, the total score and Cronbach’s α in this task were not applicable given that it is a productive task (see a similar approach in Chen, 2021).

In summary, radical identification exclusively needs learners’ orthographic ability, whereas, radical analysis requires both orthographic and graphomorphemic ability.

#### Reading Comprehension Task

This task was adopted from the reading section in the Chinese Proficiency Test (HSK)-level 4 and mainly measured learners’ general reading ability, including knowledge of vocabulary and grammar, as well as passage understanding and inference. This task included three parts: sentence reading by inserting appropriate words, sentence organizing by ordering clauses, and short-passage reading comprehension. In Part 1, the participants were asked to select an appropriate word from a vocabulary pool to insert a sentence with a blank, for example, 明天可能下雨 *你记得[ ]儿子带伞* (It might be raining tomorrow, please [] our son to bring umbrella.) The answer in this case was 提醒 [(ti) (35) (ɕiŋ) (214), “remind”]. There were 10 items in this part. In Part 2, the participants were asked to reorganize three clauses in each item and to create a new sentence. For example, Option A was “茶不仅仅是一种饮料 (Tea is not only a drink)”; Option B was “它在中国有着几千年的历史 (It has a history of several thousand years in China)”; and Option C was “而且还是一种文化 [but also (it) is a kind of culture].” The expected answer in this example was “A, C, and B.” There were also 10 items. In Part 3, the participants were asked to read several short passages ranging from 30 to 150 characters, and after each passage they were asked to select correct answers regarding the passage’s main idea and/or detailed information from four options. There were 20 questions in Part 3. Each correct selection for the items in Part 1 and 2 was calculated as 2 points, and each correct selection for the items in Part 3 as 3 points in terms of the rubrics of HSK (Hanban, 2009). The total score of the reading comprehension task was 100 points. Cronbach’s α in this task was not reported given that HSK is a national, standardized test in China, whose reliability was officially validated by Luo et al. (2011). In this study, reading comprehension task (Week 18) was selected from the published HSK tests (level 4).

### Procedures

In this study, all of the participants completed two character-level and two radical-level grapho-morphological knowledge tasks in Week 8 and one reading comprehension task in Week 18 during the first semester of the year (including 20 official weeks). The participants received a background questionnaire online, which was developed by the Office of International Students at the university when they registered for classes at the beginning of the semester. The participants completed all the paper-pencil tasks in their classrooms. Upon completion of the testing, all participants were given souvenirs for their time.

## Results

A total of 103 participants were included in the statistical analysis because two participants missed at least one task (All data and analysis results are available at: https://osf.io/avner/files/). To answer the research question posed earlier, descriptive statistics, correlational and path analyses results are presented below. The descriptive data for all the tasks are presented in Table 2, including mean scores, standard deviations (SDs), and 95% confidence intervals.

**TABLE 2:** Descriptive statistics of radical identification, radical analysis, character recognition, character manipulation, and reading comprehension (*N* = 103).

	***k***	**Mean**	**Standard deviation (SD)**	**95% Confidence interval**	
				**Lower limit**	**Upper limit**
Radical identification	24	0.52	0.26	0.47	0.57
Radical analysis	20	0.40	0.19	0.36	0.44
Character recognition	40	0.35	0.14	0.32	0.38
Character manipulation	–	6.87	3.53	6.18	7.56
Reading comprehension (Week 18)	100	0.45	0.11	0.43	0.47

*k, item number. Means, SDs, and 95% confidence interval pertain to proportion of correct choice of items in all the tasks except for the character manipulation task.*

As shown in Table 2, elementary-level learners performed better at the initial learning stage in identifying radicals (the mean accuracy rate was around 52%) than analyzing radicals (the mean accuracy rates was 40%). Second, although L2 learners did not perform well in character recognition and manipulation, relatively large SDs suggest that learners’ understanding of characters appeared individually different at least from Week 8. In addition, learners’ reading comprehension abilities in Week 18 were not satisfactory (the mean accuracy rate was 45%), but the relatively large SD indicates that some learners developed this reading ability faster, whereas, others developed it more slowly. In addition, we used Pearson product moment correlation.

coefficient to analyze the specific relevance on individual variables and intercorrelations among all the variables are reported in Table 3.

**TABLE 3:** Correlations among radical identification, radical analysis, character recognition, character manipulation, and reading comprehension (*N* = 103).

** Measures**	**1**	**2**	**3**	**4**	**5**
1. Radical identification	–	0.190∼	0.216*	0.285**	0.298**
2. Radical analysis		–	0.352***	0.489***	0.295**
3. Character recognition	.		–	0.624***	0.493***
4. Character manipulation				–	0.459**
5. Reading comprehension (Week 18)					–

∼*p* = 0.05; *p < 0.05; **p < 0.01; and ***p < 0.001.

The results in Table 3 showed that most of the observed variables were significantly and positively correlated with each other. A marginal correlation has been found between radical identification and radical analysis.

To further understand the relationships among each early character- and radical-level grapho-morphological knowledge and later reading comprehension, path analyses were conducted to investigate the direct and indirect predictions of grapho-morphological knowledge at radical- and character-levels to reading comprehension. To recapitulate, the conceptual model 1 (see Figure 1A) hypothesized that (a) radical- and character-level grapho-morphological knowledge directly predicted later reading comprehension; and that (b) radical-level grapho-morphological knowledge also made indirect predictions to later reading comprehension via character-level grapho-morphological knowledge. Second, the conceptual model 2 (see Figure 1B), based on the conceptual model 1, hypothesized that radical identification did not directly predict later reading comprehension, yet radical analysis made a direct prediction. Third, the conceptual model 3 (see Figure 1C) hypothesized that (a) character-level grapho-morphological knowledge directly predicted later reading comprehension; and that (b) radical-level grapho-morphological knowledge made indirect predictions via character-level grapho-morphological knowledge. Then, the model fits were tested separately.

**FIGURE 2:**
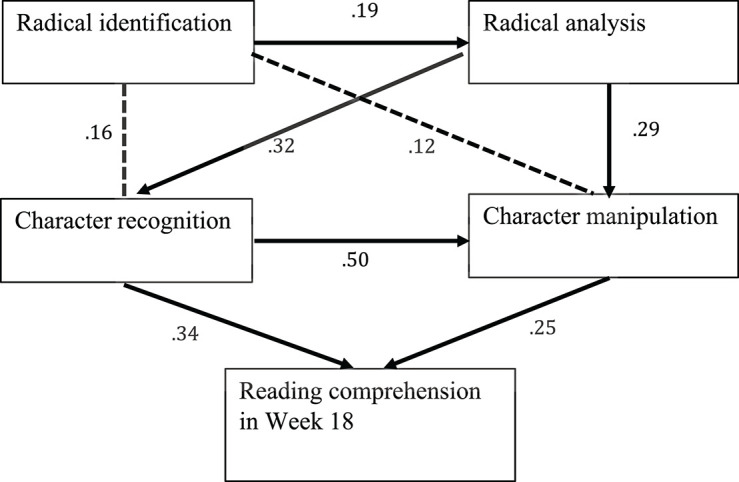
The observed path model regarding how early radical-level and character-level grapho-morphological knowledge predicts later reading comprehension.

In the conceptual model 1, a just-identified model, the degrees of freedom = 0, which means the chi-square goodness-of-fit test was not applicable. In addition, no significant direct paths were found from both radical identification and analysis to later reading comprehension (*p*s > 0.05). In the conceptual model 2, the chi-square goodness-of-fit test did not support the convergence between the conceptual model 2 and the observed model. The result does not indicate a good model fit, χ^2^ (1,103) = 3.69, *p* = 0.055 (GFI = 0.98; NFI = 0.97; CFI = 0.98; RMSEA = 0.16; χ^2^/df = 3.69). While in the conceptual model 3, the chi-square goodness-of-fit test confirmed the consistency between our hypothesized model and the observed model. The result indicates a good model fit, χ^2^ (2,103) = 4.19, *p* = 0.12 (GFI = 0.98; NFI = 0.97; CFI = 0.98; RMSEA = 0.10; χ^2^/ df = 2.10). Taken together, we used the conceptual model 3 as the hypothesized model in this study. Then, path analyses were run to investigate the direct and indirect effects of grapho-morphological knowledge at the character and radical levels on later reading comprehension (see Figure 2).

Table 4 shows the standardized regression weights of different path routes. It was found that: (a) radical identification ability marginally had a direct effect on radical analysis ($\hat{\beta }$ 0.190, *p* = 0.05), character recognition ($\hat{\beta }$ 0.155, *p* = 0.10) and character manipulation ($\hat{\beta }$ 0.123, *p* = 0.09), and radical analysis ability significantly and directly contributed to character recognition ($\hat{\beta }$ 0.322, *p* < 0.001) and character manipulation ($\hat{\beta }$ 0.291, *p* < 0.001). (b) Character recognition ability directly had significant impacts on character manipulation ability and reading comprehension in Week 18 ($\hat{\beta }$ 0.495, *p* < 0.001;$\hat{\beta }$ 0.338, *p* < 0.01); character manipulation ability significantly predicted reading comprehension in Week 18 ($\hat{\beta }$ 0.248, *p* < 0.05).

**TABLE 4:** Standardized regression weights for all measures based on conceptual model 3.

**Paths**	$\hat{\beta }$	**SE**	**CR (z)**	***p***
Radical A ← Radical I	0.190	0.058	1.956	0.050
Character R ← Radical I	0.155	0.084	1.660	0.097
Character R ← Radical A	0.322	0.142	3.462	<0.001
Character M ← Radical I	0.123	0.041	1.674	0.094
Character M ← Radical A	0.291	0.072	3.808	<0.001
Character M ← Character R	0.495	0.048	6.438	<0.001
Reading C ← Character R	0.338	0.203	3.147	0.002
Reading C ← Character M	0.248	0.327	2.303	0.021

*radical I, radical identification ability; radical A, radical analysis ability; character R, character recognition ability; character M, character manipulation ability; reading C, reading comprehension in Week 18.*

Table 5 shows the direct and indirect effects of grapho-morphological knowledge at the character and the radical levels on reading comprehension in Week 18. The findings indicated that (a) radical-level grapho-morphological knowledge significantly made indirect contributions to later reading comprehension via other reading subskills at the relatively higher levels. Specifically, radical identification ability via radical analysis ability indirectly contributed to later reading comprehension. Subsequently, radical analysis ability had an indirect contribution to later reading comprehension via character-level knowledge; and (b) character-level grapho-morphological knowledge significantly made direct and indirect contributions to later reading comprehension. Specifically, character recognition ability directly contributed to reading comprehension in Week 18, as well as indirectly contributed to reading comprehension in Week 18 through character manipulation ability; character manipulation ability directly predicted reading comprehension in Week 18.

**TABLE 5:** Standardized total effects, direct effects, and indirect effects on reading comprehension based on conceptual model 3.

**Reading comprehension in Week 18**	**Direct effect**	**Indirect effect**	**Total effect**
Radical I	–	0.144**	0.144**
Radical A	–	0.221**	0.221**
Character R	0.338**	0.122*	0.461**
Character M	0.248*	–	0.248*

radical I, radical identification ability; radical A, radical analysis ability; character R, character recognition ability; character M, character manipulation ability. *p < 0.05 and **p < 0.01.

To answer the research question, early grapho-morphological knowledge at both levels significantly affected later reading comprehension in L2 Chinese; character-level grapho-morphological knowledge made both direct and indirect contributions to later reading comprehension in L2 Chinese while radical-level grapho-morphological knowledge made indirect contributions.

## Discussion

This study examined the relations between early grapho-morphological knowledge and later reading comprehension in beginning-level collegiate L2 Chinese learners, and measured grapho-morphological knowledge at both character and radical levels. First, the findings indicated that grapho-morphological knowledge at the character and radical levels in Chinese, particularly for beginning-level L2 learners, are both significant predictors of reading comprehension across time. Previous L1 Chinese studies have shown that grapho-morphological knowledge (at the character level) were unique and significant predictors of reading comprehension in Chinese (e.g., Ku and Anderson, 2003; Shu et al., 2006; Yeung et al., 2013; Wu, 2017; Zhang, 2017; Zhang and Koda, 2018), and the reading knowledge had long-term effects for native Chinese speakers (e.g., Pan et al., 2016; Zhang, 2016). This study not only supports the positive roles of character-level grapho-morphological knowledge in later reading comprehension, but also expands our understanding that early radical-level grapho-morphological knowledge could play significant, predictive roles in later reading comprehension. Tables 4, 5 showed joint predictions of early grapho-morphological knowledge at the character and radical levels in later reading comprehension. The results suggest that, in addition to beginning-level learners’ character (morpheme) knowledge, L2 learners’ sensitivity to character-internal structures and their understanding to the compositional rules at the character and radical levels could facilitate their reading development in Chinese (Chen and Feng, 2020).

Second, early radical-level grapho-morphological knowledge indirectly predict later reading comprehension via their relatively higher-level grapho-morphological knowledge (at the character level). Admittedly, radical-level grapho-morphological knowledge is about learners’ orthographic knowledge of characters, radicals’ semantic representations, and their understanding of how to compose radicals and simple characters in a systematic writing rule; and a better understanding of character-internal structures and their semantic categories might not give L2 learners advantages when determining the meaning of a phrase, sentence or short passage. However, this better understanding helps L2 learners, particularly at the early stage of learning L2 characters, more easily read, analyze, and memorize characters and multicharacter words (Shen and Ke, 2007; Tong and Yip, 2015). Therefore, learners with more sensitivity to character-internal structures, as well as radical positions and semantic categories, often have a larger character and vocabulary size and a deeper understanding of characters’ meanings and collocations. The character and multicharacter word size (numbers of known characters and words) and depth (how well they know each character and word) (cf. Koda, 2005) directly affect leaners’ word-meaning inference and sentence-meaning construction in Chinese (e.g., Zhang, 2015, 2016; Zhang and Yang, 2016).

Consistent with the previous studies (e.g., Shen and Ke, 2007; Lü et al., 2015), the findings in this study support the direct contribution of radical-level grapho-morphological knowledge to character recognition and manipulation abilities. The results also indicated that the radical-level grapho-morphological knowledge, even for the radical identification subskill at the lowest level in the Chinese hierarchical writing system, positively and indirectly affect and predict later reading comprehension at the highest level. This indirect contribution of the radical-level grapho-morphological knowledge can explain why the significant predictive value of radical awareness for reading comprehension was not found in Zhang et al. (2012) study. In Zhang et al.’s study, they used the regression analysis to treat the reading subskills at the radical and character levels as a group, but did not investigate the indirect effects of the radical-level knowledge on reading comprehension.

Finally, this study has found that early character-level grapho-morphological knowledge directly and indirectly predicts later reading comprehension. The general findings were consistent with previous studies for native Chinese speakers (e.g., Pan et al., 2016; Zhang, 2016), though the involved reading subskills were different. In fact, L2 Chinese learners unsurprisingly rely more on character-level knowledge in sentence-meaning and text-meaning comprehension, because characters, the basic units, were closely related to morphemes and words in the Chinese writing system (Zhu, 1982). One interesting finding in this study shows that the predictive role of character manipulation ability relatively strengthens in reading comprehension as learning-duration increases. This ability to create new words using known characters, to a larger extent, depends on learners’ vocabulary knowledge (size and depth), which is a reading subskill at a higher level (the multicharacter level) than the character level. One possibility is that some learners gradually integrate reading subskills at the lower levels and can more fully use reading subskills at the higher levels in reading comprehension, as they are more familiar with the Chinese writing system. Yet, further replication research is needed in the future.

## Conclusion, Limitations, and Implications

In summary, this study investigated the relations among grapho-morphological knowledge at the character and subcharacter/radical levels in Week 8 and later reading comprehension in Week 18 for beginning-level collegiate L2 Chinese learners. The main findings showed that grapho-morphological knowledge both at the character and radical levels were significant predictors of later reading comprehension. Path analyses results provided the specific, direct and indirect paths among character- and radical-level grapho-morphological knowledge to later reading comprehension. Specifically, early character recognition and manipulation directly and indirectly predicted later reading comprehension; in addition, early radical identification and analysis indirectly predicted later reading comprehension through character recognition and manipulation.

While this study has provided new empirical evidence, there were some limitations. First, only character recognition and manipulation at the character level were involved in this study. Other key reading abilities at the character and higher levels such as phonological awareness, and vocabulary knowledge, as well as cognitive abilities such as non-verbal reasoning, should be considered in the future. Second, the interactions of L1 effects and L2 reading subskills have not been considered. A future study would be improved if the participants’ L1 backgrounds were controlled with a larger sample size for a longer period (e.g., 1 year or more). Participants with the same L1 could minimize the possible effects brought by different L1 backgrounds and L1-L2 linguistic distance (e.g., Koda, 2005, 2007, 2015). A longer observation (e.g., more than 1 year) with data gathered at multiple times points in a larger group would provide more convincing data.

Pedagogically, the empirical findings in this study not only reemphasize the importance of early character knowledge in reading Chinese, but also provide new evidence of the predictive roles of subcharacter/radical-level knowledge. It has been widely acknowledged that native speakers should memorize as many new characters as possible at the early stage of Chinese learning; however, no consensus has been reached regarding how to more efficiently instruct L2 learners in character learning. Given the roles of radical-related reading subskills, it is suggested that instructions and exercises related to character-internal structures and radicals’ roles should receive more attention. Identifying character-internal structures can help L2 Chinese learners who are unfamiliar with the Chinese writing system gradually understand the basic rules of how radicals compose characters. Learners who have a deeper understanding of compositional rules of characters have less memory burden when they remember new characters. Similarly, learners who know more characters and compositional rules of words more easily memorize new words, particularly for those composed of familiar characters. Learners’ Chinese reading comprehension builds on both character- and subcharacter-level reading subskills. Therefore, a combination of character-based and radical-based character learning in an L2 Chinese class may be considered, particularly at the initial stage of learning an L2. Nevertheless, emphasizing the importance of grapho-morphological knowledge at the initial period of learning Chinese does not mean that such corresponding instruction is the only important aspect in teaching and learning L2 Chinese reading. Reading development in L2 may also be expanded by recognizing various factors in learning to read, such as prior knowledge, reading strategies, extensive reading experience, and personal interest.

## Data Availability Statement

The raw data supporting the conclusions of this article will be made available by the authors, without undue reservation.

## Ethics Statement

The studies involving human participants were reviewed and approved by Carnegie Mellon University. The patients/participants provided their written informed consent to participate in this study.

## Author Contributions

TC: responsible for manuscript writing, data collection, and data analysis. SK: responsible for literature review and discussion. KK: responsilbe for the framework and discussion. All authors contributed to the article and approved the submitted version.

## Conflict of Interest

The authors declare that the research was conducted in the absence of any commercial or financial relationships that could be construed as a potential conflict of interest.

## Publisher’s Note

All claims expressed in this article are solely those of the authors and do not necessarily represent those of their affiliated organizations, or those of the publisher, the editors and the reviewers. Any product that may be evaluated in this article, or claim that may be made by its manufacturer, is not guaranteed or endorsed by the publisher.
